# Topical R1 and R2 Prophylactic Treatment of Acute Radiation Dermatitis in Squamous Cell Carcinoma of the Head and Neck and Breast Cancer Patients Treated With Chemoradiotherapy

**Published:** 2015-06-24

**Authors:** Ana Manas, Miguel Santolaya, Violeta Mirela Ciapa, Belén Belinchón, Frances Tully

**Affiliations:** ^a^Radiation Oncology Department, La Paz University Hospital, Madrid, Spain; ^b^Foundation for Biomedical Research, Radiation Oncology Department, La Paz University Hospital, Madrid, Spain; ^c^Nursing Unit, IMO Group, Madrid Institute of Oncology, Madrid, Spain; ^d^Water-Jel Europe LLP, Unit 3&4, The Mead Business Centre, Hertford, United Kingdom.

**Keywords:** radiation dermatitis, chemoradiotherapy, head and neck cancer, breast cancer, prophylaxis

## Abstract

**Objective:** A clinical study was conducted on the use of the topical Lactokine-based R1 and R2 system as a prophylactic treatment of acute radiation dermatitis in patients with squamous cell cancer of the head and neck and breast cancer treated with chemoradiotherapy. **Methods:** Ninety-eight patients were studied who attended the Radiation Oncology Services, La Paz University Hospital, Madrid, for treatment with chemoradiotherapy for head and neck cancer (*n* = 19) and breast cancer (*n* = 79). The treatment group (R1 and R2) included 51 patients; 47 control patients were given the local standard topical treatment (5% wt/wt urea lotion). At 3 postradiotherapy follow-up clinics, radiation dermatitis was graded, if present. All patients were administered with “quality-of-life” questionnaires. **Results:** Treatment with R1 and R2 significantly reduced the grade of radiation dermatitis in patients treated with chemoradiotherapy. At the fourth (last) clinic visit, at 2 weeks following the end of radiation treatment, 66.7% of patients in the treated group (R1 and R2) were free from radiation dermatitis compared with 34% in those given the center's usual skin care (topical urea lotion). There were no reported side effects, and quality of life improved for patients treated with R1 and R2. **Conclusion:** Topical skin treatment with the R1 and R2 system has been shown to be effective in preventing, reducing the onset, and reducing the degree (grade) of radiation dermatitis in head and neck and breast cancer patients treated with chemoradiation.

Radiation dermatitis is a common skin toxicity associated with radiation therapy for cancer and affects up to 95% of patients.^1^ Acute radiation dermatitis is of particular importance in patients treated for head and neck cancer and breast cancer, because of the high doses of radiation given to the skin during treatment.^2^

The standard treatment of early breast cancer includes surgery, chemotherapy, and radiotherapy. Chemoradiotherapy (CRT) may produce a biological synergy effect that increases the efficacy of treatment.^3^ Different parts of the body have different sensitivities to radiation, with the chest and the neck being the most sensitive. Surgery to the breast, including breast reconstruction, makes the breast skin more sensitive to radiation damage.^2^^,^^4^

Squamous cell carcinoma of the head and neck (SCCHN) is the most common primary malignancy of the upper aerodigestive tract.^5^ Combined chemotherapy and radiotherapy has been shown to increase patient survival and in many cases to reduce the extent of surgical resections.^5^

In chemotherapy regimens, traditional platinum chemotherapy agents include cisplatin, carboplatin, and oxaliplatin. However, epidermal growth factor receptor (EGFR) inhibitors are associated with an increase in skin toxicity.^6^ Other clinical risk factors for radiation dermatitis include increasing age, actinic skin damage, obesity, smoking, and connective tissue disease.^2^

The skin changes of an acute radiation injury can occur within hours or days of radiation treatment but usually commence within 1 to 4 weeks.^2^ This acute phase of radiation injury is due to structural tissue damage, generation of free radicals, and damage to nuclear and mitochondrial DNA; there may also be an inflammatory response.^2^ Chronic or repeated exposure to ionizing radiation induces vascular damage and dermal fibrosis (scarring).^2^ The symptoms of radiation dermatitis, including discomfort and pain, can have a profound effect on the quality of life (QOL) and may cause termination of radiotherapy treatment before it has been completed.^2^ At this time, there is no “gold standard” measure of subjective, patient-rated symptoms associated with radiation dermatitis or its effects on patient QOL.^7^

The histopathological changes in the skin associated with radiation injury are well documented, but, in the clinic, radiation dermatitis is graded visually.^8^ Clinical grading systems for radiation dermatitis include the National Cancer Institute Common Toxicity Criteria–Adverse Event (CTCAE or CTC grades) and the Radiation Therapy Oncology Group toxicity scoring system.^8^^-^10 Acute radiation dermatitis includes edema, erythema, hair loss, pigment changes, “dry desquamation,” and “moist desquamation.”^8^^-^10

Currently, there is no recognized single dermatological prophylactic “standard of care” for patients undergoing high-dose radiotherapy treatment.^11^^,^^12^ In 2006, the Supportive Care Guidelines Group evaluated the methods to prevent acute skin reactions (within the first 6 months) of radiation therapy and to determine the optimal methods to treat acute skin reactions.^13^ The study recommendations included the initial use of a plain, nonscented, lanolin-free hydrophilic cream as a preventive measure and treatment with low-dose (1%) corticosteroid cream to reduce itching and irritation.^13^ The use of topical urea lotion (3%–40%) has been adopted as a prophylactic treatment of radiation dermatitis in many clinical centers, including our own.^14^

In 2013, Wong and colleagues^15^ systematically reviewed 56 studies supporting guidelines for interventions to prevent or treat radiation dermatitis. Of these studies, 45 randomized controlled clinical trials addressed prevention of acute radiation dermatitis and 9 addressed treatments.^15^ Most studies of interventions to prevent or treat radiation dermatitis were based on insufficient evidence to support or refute their use.^15^

The topical Lactokine-based R1 and R2 system, manufactured by Water-Jel Technologies (Carlstadt, NJ), is also marketed in the United States as the Radiaderm Advanced Skincare System.^16^ The R1 and R2 system was initially classified by the US Food and Drug Administration as a medical device for use in the prevention of radiation dermatitis; from 2010, it was approved as a class IIa medical device under the 93/42/EEC (European Economic Community) Medical Device Directive (CE554803) and the 2010 Directive (007/47/EC).^16^

The R1 and R2 system consists of 2 parts: R1 is a water-based gel and R2 is a skin-moisturizing lotion that contains Lactokine, a protein derived from milk. Lactokine Fluid (manufactured by CLR–Chemisches Laboratorium, Berlin, Germany) conforms to the 67/548/EEC safety specifications and contains between 1% and 5% milk protein with lactose and minerals.^16^

In 2013, a case report was published on the benefits of the prophylactic use of the R1 and R2 system in a 63-year-old female patient with a squamous cell carcinoma of the hypopharynx undergoing platinum-based CRT.^17^ At the same time, these authors launched the first prospective clinical trial on the prophylactic use of R1 and R2 in patients with SCCHN receiving CRT (CREAM-1).^18^ Preliminary findings showed that the topical application of cooling R1 gel and moistening R2 lotion was feasible, safe, and effective in prophylactic treatment of acute radiation-induced dermatitis in this patient group.^19^ This trial is now completed, and the final results are awaiting analysis.

The purpose of this study was to evaluate the R1 and R2 system in the prevention or alleviation of radiation dermatitis in the clinical setting of an academic radiation oncology department. Two groups of patients were studied who are known to be most susceptible to radiation dermatitis: breast cancer patients and patients with SCCHN.

## PATIENTS AND METHODS

This study was conducted on 98 patients who were treated at the Radiation Oncology Services, La Paz University Hospital, Madrid. The patients included those scheduled for chemoradiation (CRT) therapy for SCCHN (*n* = 19; 16M:3F) and breast cancer (*n* = 79).

Patients were recruited and randomly assigned consecutively to the “treatment” and “control” groups. Control patients were assigned to our usual standard skin care treatment, containing 5% wt/wt urea (*n* = 47; 9M:38F) and to R1 and R2 topical treatment (*n* = 51; 7M:44F). The initial study sample included 102 patients; 4 patients were excluded from the study because they did not meet the study criteria or did not complete CRT treatment.

### Ethics statement

This study was approved by the Radiation Oncology Service and Clinical Research Ethics Committee of La Paz University Hospital (Madrid, Spain) (registration no. HULP: 3528). Written informed consent was obtained from all patients prior to the start of the study.

### Study schedule

Each patient participated in the study for approximately 3 months, which included a 1-week screening period, 5 to 8 weeks of treatment, and follow-up clinic attendance. Four clinic visits took place, from a week before the start of radiation therapy, to evaluate each patient (first clinic visit), to 2 weeks after the end of radiation therapy (fourth clinic visit). The second clinic follow-up clinic visit and assessment were at 4 weeks following the start of radiation treatment. The third clinic follow-up visit and assessment were at the end of radiation treatment. The fourth and final clinic follow-up visit and assessment were 2 weeks following the end of radiation treatment. The total length of the study was approximately 10 weeks for breast cancer patients and 12 weeks for patients with SCCHN.

At each clinic visit, patient information was collected relating to radiation-associated skin changes, physical examination, and assessment of QOL. Analysis of patient data and grading of radiation dermatitis, if present, were done at 3 of the follow-up clinic visits, the second, third, and fourth (final) follow-up clinic visits (Table 1).

### Primary and secondary study objectives

The primary objective of this study was to evaluate whether there was a reduction in progression to severe (CTC grades 3 and 4) radiation dermatitis with the use of topical R1 and R2 treatment.

The secondary objectives of the study were, first, to evaluate the overall response rate and, second, to evaluate the effects on QOL, if any, with the use of topical R1 and R2 treatment. The QOL assessment was done using EORTC (European Organization for Research and Treatment of Cancer) patient questionnaires.^20^^-^22

### Patient inclusion criteria

Patients with histologically confirmed, localized (nonmetastatic) SCCHN and breast cancer were recruited. Patients were about to commence standard fractionated radiotherapy while receiving platinum-based chemotherapy and with the treatment duration of between 6 and 8 weeks. Patients were 18 years and older, with ECOG (Eastern Cooperative Oncology Group) Performance Functional Status of 0 to 2, a life expectancy of 6 months or more, and provided signed informed consent.

### Patient exclusion criteria

Patients were excluded if they had distant metastases, previous radiotherapy for SCCHN or breast cancer, ongoing participation in any other clinical study or trial, or concomitant treatment with an EGFR inhibitor. Patients who were pregnant or breast-feeding were excluded from the study, as were those with known hypersensitivity to any of the R1 and R2 treatment components. Patients with previous or concurrent cancer within 5 years of starting the study and any other social or medical conditions that would affect participation in or evaluation of the study were excluded.

### R1 and R2 treatment

Topical R1 was applied once a day within 2 hours after radiation therapy. R2 was applied 4 times a day, 3 times during the daytime and a last application just before retiring to bed. R1 and R2 skin treatment was applied from the first day of radiation treatment until 2 weeks after the end of radiation treatment.

### Control treatment

The standard of care for radiation dermatitis at Radiation Oncology Services, La Paz University Hospital, is to use a urea-containing ointment.^14^ In the control arm of the study, the urea-containing emollient, Vitalur NM (Nutricion Medica S.L., Madrid, Spain) containing 5% wt/wt urea, was applied from day 1 of radiation treatment until 2 weeks after the end of radiation treatment.

### CTC grading of radiation dermatitis

**Figure 1:**
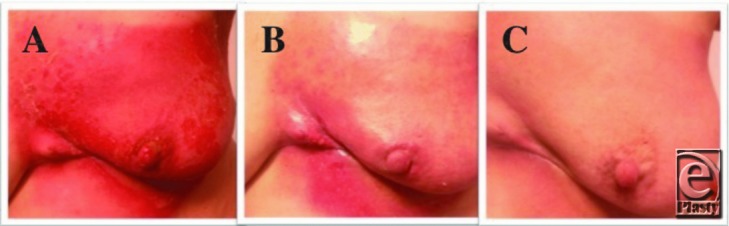
Grading of radiation dermatitis during chemoradiation of the breast. Appearance of the breast skin in a (R1 and R2) treated patient receiving radiation therapy for breast cancer, at sequential follow-up clinic visits. (a) Skin of the breast in the radiation field showing radiation dermatitis, CTC grade 2. This photograph shows “moist desquamation.” (b) Skin of the breast in the radiation field showing radiation dermatitis, CTC grade 1. (c) Skin of the breast in the radiation field with a normal appearance, CTC grade 0. CTC indicates Common Toxicity Criteria.

The grade of radiation dermatitis was assessed using the National Cancer Institute CTC classification (V.4.03).^8^^-^10 Photographic documentation of the skin lesions was performed (Fig 1). The following describes the appearances of the skin for each grade:

*Mild radiation dermatitis, CTC grade 1*: The onset is within days to weeks of commencing therapy, but symptoms may fade within a month. The skin shows either mild erythema that blanches on compression or “dry desquamation.” Pruritus and hair loss may be seen (Fig 1*b*).
*Moderate radiation dermatitis, CTC grade 2*: This may be most severe 1 to 2 weeks after the end of treatment. The skin shows intense edema and erythema that is painful, and this may progress to loss of the epidermis and to “moist desquamation” that includes fibrinous exudates and bullae (Fig 1*a*).
*Severe radiation dermatitis, CTC grade 3*: This form of dermatitis includes confluent moist desquamation that may progress to full-thickness skin necrosis and ulceration. Pain may be quite severe.
*Severe radiation dermatitis, CTC grade 4*: This is a rare form of dermatitis, occurring in less than 5% of patients, and includes full-thickness skin necrosis and ulceration. It may require discontinuation of radiation therapy and treatment with surgical debridement, full-thickness skin graft, or reconstructive surgery.

### QOL questionnaires

All patients were given EORTC QOL questionnaires, including (QLQ) C30 (30 questions) (version 3), (QLQ) Head and Neck (H&N) C30 (65 questions) (version 3), and Questionnaire (QLQ) Breast Cancer Br23 (53 questions).^20^^-^22 Questionnaires were completed in every follow-up clinic visit from the initial screening day (first clinic visit) to 2 weeks posttreatment (fourth clinic visit).

## RESULTS

**Figure 2:**
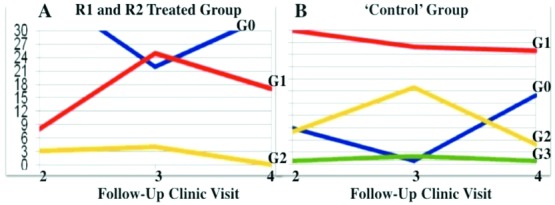
Diagram to compare the progression of CTC grades of radiation dermatitis at the second, third, and fourth follow-up clinic assessments in (a) treated (R1 and R2) patients and (b) control patients. The number of patients at the second, third, and fourth follow-up clinics is presented according to CTC grade of radiation dermatitis: G0, grade 0; G1, grade 1; G2, grade 2; G3, grade 3.^8^^-^10 CTC indicates Common Toxicity Criteria.

The application of R1 and R2 topical skin treatment was well tolerated, with no reported side effects. Although the clear differences in CTC grading responses between the treated and control patient groups allow for direct comparison of data, formal hypothesis tests are provided later. The original data are presented in Table 2 and Figure 2.

### Analysis of the onset of radiation dermatitis in the treatment group (R1 and R2) and the control group

The comparison of the 2 groups was done by testing the hypotheses that the probability of a patient having acute radiation dermatitis is greater under the treatment group than under the control group at the different clinic visits. The rejection of these hypotheses at standard significance levels (*P* ≤ .01) supports the use of R1 and R2 treatment. The *P* values presented later were obtained using the Wald test for the difference in proportions.^23^

At the second follow-up visit, 50 patients presented with acute radiation dermatitis; 22% (11/51) in the R1 and R2 treatment group and 78% (39/47) in the control group. This is a significant difference (*P* < .0001).

At the third follow-up visit, 76 patients presented with acute radiation dermatitis; 57% (29/51) in the R1 and R2 treatment group and 100% (47/47) in the control group. This is a significant difference (*P* < .0001).

At the fourth follow-up clinic visit, 48 patients presented with acute radiation dermatitis: 33.3% (17/51) in the R1 and R2 treatment group and 66% (31/47) in the control group. This is a significant difference (*P* = .0003). At this fourth (final) follow-up visit, 66.7% (34/51) of patients in the R1 and R2 treatment group were free of radiation dermatitis. In contrast, 34% (16/47) of patients in the control group were free of radiation dermatitis.

Overall, fewer patients had acute radiation dermatitis in the treatment group than in the control group at the different follow-up clinic visits, and these findings are statistically significant.

### Analysis of the severity (grade) of radiation dermatitis in the treatment group (R1 and R2) and the control group

In the R1 and R2 treatment group, 25% (13/51) of patients who were free of radiation dermatitis at the first follow-up visit did not develop radiation dermatitis at any subsequent visit.

In the control group, at the fourth follow-up visit, of the 31 patients who continued to have radiation dermatitis, 7 patients were CTC grade 2 and the rest, 24 patients, were CTC grade 1. In this study, there were no CTC grade 4 radiation dermatitis cases in either group.

### Risk analysis for the prevention of radiation dermatitis in the treatment group (R1 and R2) and the control group

We then used risk ratios to test the hypotheses that the probability of a patient with acute radiation dermatitis is greater under the treatment group than under the control group at the different clinic visits. Those risk ratios being significantly smaller than a value of 1.0 support the use of R1 and R2 treatment. The *P* values presented later are obtained using the Wald test for the ratio of proportions.

At the second follow-up visit, patients belonging to the R1 and R2 treatment group were 0.26 times as likely to have developed radiation dermatitis compared with patients belonging to the control group. This is a significant difference (*P* < .0001).

At the third follow-up visit, patients belonging to the R1 and R2 treatment group were 0.57 times as likely to have developed radiation dermatitis compared with patients belonging to the control group. This is a significant difference (*P* < .0001).

At the fourth follow-up visit, 2 weeks after the end of radiation treatment, patients belonging to the R1 and R2 treatment group were 0.5 times as likely to be suffering from radiation dermatitis compared with patients belonging to the control group. This is a significant difference (*P* = .0012).

### Assessment of QOL in the treatment group (R1 and R2) and the control group

All the patients completed the EORTC QOL questionnaires at each follow-up clinic. Comparison of the responses between the treatment group (R1 and R2) and the control group showed a difference in the number of negative responses in the control group. However, the most relevant questions for patients receiving R1 and R2 treatment related to skin dryness, stinging, and desquamation for patients with breast cancer and the reduced use of medication for pain control in patients with SCCHN.

### Summary of findings

These findings have shown that treatment with R1 and R2 in patients receiving CRT for breast cancer and SCCHN had the following beneficial outcomes when compared with standard skin treatment (5% wt/wt urea lotion):

1. R1 and R2 treatment prevented the development of acute radiation dermatitis in 25% of patients.
2. R1 and R2 treatment delayed the onset of radiation dermatitis in 60% of patients.
3. R1 and R2 treatment reduced the severity (CTC grade) of radiation dermatitis by 50% in those patients who developed it.
4. R1 and R2 treatment improved the subjective assessment of patients’ QOL.

## DISCUSSION

The majority of patients receiving radiation therapy will suffer from some degree of radiation dermatitis.^1^ As yet, there have been no published randomized, controlled, clinical trials to evaluate the most effective preventive treatments, and there is little evidence-based guidance available to radiotherapists, oncologists, dermatologists, and oncology nursing staff.

We have presented the first comparative study of the use of R1 and R2 in a treated group and a nontreated group of patients receiving CRT. In this study, we evaluated the 2 groups of patients receiving radiotherapy who are most susceptible to severe radiation dermatitis: patients receiving treatment of SCCHN and patients receiving treatment of breast cancer. However, the study population was predominantly breast cancer patients (79/98; 80.6%) rather than patients with SCCHN. In future, larger studies, the surgical history of patients treated with CRT for breast cancer could be assessed in relation to radiation dermatitis, including any history of breast implants in breast reconstruction surgery. Postsurgical breast skin is susceptible to radiation toxicity, as is the skin overlying breast implants, possibly due to lack of dissipation of heat away from the breast.^4^

The clinical effects of radiation skin toxicity or radiation dermatitis affect the QOL for cancer patients.^2^ EORTC QOL questionnaires were used in this study to provide information on whether R1 and R2 treatment affected symptoms and other QOL factors for CRT-treated patients. However, these are general oncology questionnaires and are not specific to the evaluation of dermatological symptoms. For future studies, questionnaires could be specifically designed to evaluate radiation dermatitis symptoms. An example of such a specific study questionnaire is the Nordic Occupational Skin Questionnaire (NOSQ-2002) designed to collect data on occupational eczema.^24^ An objective and subjective evaluation of radiation dermatitis could be combined to include patient and treatment parameters, observer scoring, and patient-reported symptoms, as in the Skin Toxicity Assessment Tool, developed by Berthelet and colleagues^25^ in 2004.

This study evaluated patients treated with platinum-based CRT and excluded patients treated with EGFR inhibitors, which are known to result in skin reactions.^6^ Platinum-based chemotherapy and chemotherapeutic agents of any kind may be associated with some degree of skin toxicity.^26^ However, it would be difficult to select patients receiving radiation treatment as a monotherapy that also involves the irradiation of the skin.

For future studies, recruiting CRT-treated patients at national and international centers would provide a larger study population. Larger studies would allow for analysis of CRT treatment subgroups or cofactors associated with either the severity of radiation dermatitis or the response to R1 and R2 therapy. These factors could include treatment response by age, sex differences, smoking history, ethnicity, previous sun exposure, tumor stage, and type and dosage of chemotherapy.^1^^,^^2^^,^^5^

Although our initial study has been limited in size, we believe that our findings would justify further larger comparative treatment studies. Randomized, controlled, multicenter clinical trials are long overdue in the field of radiation dermatitis prophylaxis to allow for the development of evidence-based clinical guidelines. Only when this clinical evidence becomes available can the prophylactic therapy of this common and distressing condition be standardized for cancer patient care.

## CONCLUSION

As yet, there are no evidence-based clinical guidelines for the prophylaxis and treatment of radiation dermatitis.^27^ The R1 and R2, Lactokine-based, topical agent has been described previously in a case report as a potential prophylactic treatment option.^17^ The results are awaited of a controlled clinical evaluation of R1 and R2 as a topical skin treatment in patients wit SCCHN.^17^ We have reported the findings of the first study to evaluate R1 and R2 in the prevention and alleviation of radiation dermatitis in a controlled study in the clinical setting of a radiation oncology service.

This study, of patients receiving CRT therapy for SCCHN and breast cancer in a single radiation oncology center, supports the use of R1 and R2 as a safe and effective topical skin treatment. R1 and R2 treatment has been shown to prevent or delay the onset of radiation dermatitis, to reduce the severity of radiation dermatitis, and to contribute to patient QOL during radiation treatment.

## Figures and Tables

**Table 1:** Study schedule and patient monitoring clinic visits before, during, and after CRT

Patient evaluation	First clinic visit: 7 d before the start of CRT	Second clinic visit: 3–4 wk after the start of CRT	Third clinic visit: at the end of CRT	Fourth clinic visit: 2 wk following the end of CRT
Written informed consent	✓			
Demographic data (age, sex, tumor type, grade, stage)	✓			
Medical history and physical examination	✓			
CTC grading of radiation dermatitis^8^^-^10 (and photography)		✓	✓	✓
Completion of EORTC quality-of-life questionnaires^20^^-^22		✓	✓	✓

CRT indicates chemoradiotherapy; CTC, Common Toxicity Criteria; EORTC, European Organization for Research and Treatment of Cancer.

**Table 2:** Summary of the CTC grades of radiation dermatitis in the treated and control patients at the second, third, and fourth follow-up clinic visits

CTC grade (0–3)	R1 and R2 treatment group	Control group	Total
**Second follow-up clinic visit**			
**0**	40	8	**48**
**1**	8	32	**40**
**2**	3	7	**10**
**3**	0	0	**0**
**Total**	**51**	**47**	**98**
**Third follow-up clinic visit**			
**0**	22	0	**22**
**1**	25	28	**53**
**2**	4	18	**22**
**3**	0	1	**1**
**Total**	**51**	**47**	**98**
**Fourth follow-up clinic visit**			
**0**	34	16	**50**
**1**	17	27	**44**
**2**	0	4	**4**
**3**	0	0	**0**
**Total**	**51**	**47**	**98**

CTC indicates Common Toxicity Criteria.
